# Editorial: Endocrine disruptors in molecular and structural endocrinology

**DOI:** 10.3389/fendo.2025.1561253

**Published:** 2025-02-10

**Authors:** Julianne M. Hall

**Affiliations:** Frank H. Netter MD School of Medicine, Quinnipiac University, North Haven, CT, United States

**Keywords:** endocrine disrupting chemical, PPARγ, obesity, screening tools, bisphenol A

Endocrine disrupting chemicals (EDCs) are natural or synthetic agents that persist in our environment. EDCs manifest deleterious effects on normal endocrine physiology by mimicking or blocking endogenous hormone functions. Humans and wildlife experience vast exposure to EDCs through food, water, air, soil, and industrial and household products. Some of the most prevalent EDCs include pesticides, pharmaceuticals, food packaging agents, household products, and plant-derived phytoestrogens. There are numerous molecular and structural mechanisms by which EDCs interfere with endogenous hormones. Specifically, EDCs can alter hormone synthesis, metabolism, and receptor expression; activate or inhibit hormone receptors; alter cellular signaling; cause inappropriate cell growth or differentiation; modify intracellular, cellular, and tissue structure and composition; and induce genomic modifications. This Research Topic provides new insights into the role of EDCs in altering endocrine function at the molecular and structural levels and informs about new methods for screening for toxicological effects.

EDCs play a pivotal role in altering metabolism, energy balance, appetite, and adiposity. Numerous epidemiological studies indicate a causal relationship between EDC exposure and obesity, and these agents are now appropriately referred to as ‘environmental obesogens.’ Bisphenol A (BPA), a persistent chemical found in household, industrial, and medical products is an established obesogen, as levels detected in humans are associated with increased adiposity.

While the manufacturing of BPA-free products has become more prevalent, the compounds that replace it, Bisphenol S (BPS), Bisphenol F (BPF), and Bisphenol AF (BPAF), have endocrine disrupting activities. In this Research Topic, Varghese and Hall highlighted the current mechanistic and epidemiological data on BPA substitutes and their potential obesogenic properties. BPA substitutes have been detected in the majority of human urine samples worldwide, and elevated levels are significantly associated with obesity and other indices of metabolic disease. Using established cellular and animal models of adipogenesis, pro-obesogenic effects of BPS and BPF were identified, and these actions involved activation of the nuclear receptor peroxisome proliferator-activated receptor γ (PPARγ) and upregulation of PPARγ-regulated adipogenic gene expression. It was also revealed that the obesogenic effects of BPS are more pronounced than those observed for BPA during both prenatal and postnatal exposure. These observations raise the question of whether public health measures should be invoked to regulate exposure to BPA substitutes as has been done for BPA.

In addition to PPARγ, several other nuclear receptors, including the structurally related PPARα, are involved in the regulation of energy utilization. Whereas PPARγ activation is associated with insulin sensitization and adipocyte differentiation, PPARα reduces triglyceride levels by enhancing fatty acid oxidation and is involved in the regulation of energy homeostasis. Numerous EDCs are known to alter the activities of PPARα and PPARγ. However, current international Organization for Economic Co-operation and Development (OECD) chemical regulatory testing methods lack screening protocols to detect agents with obesogenic activities. The development of such procedures would require the identification of reference chemicals for validation and test controls. Ozcagli et al. compiled a comprehensive review and evaluation of chemicals that may be suitable for the development of PPARα and PPARγ activation assays and white adipose tissue adipogenic assessments. The criteria for selecting chemicals for evaluation were based on the possession of the necessary structural and physico-chemical properties that enable interaction with PPARs and the ability to alter their transcriptional activities and subsequent metabolic actions. The authors identified 41 chemicals as a source list, with numerous EDCs such as BPA, phthalates, and pesticides (DDT), along with known PPARα and γ agonists and antagonists. This work provides a significant contribution to the OECD guidelines, allowing for improved screening and detection of potential obesogenic activities of chemicals to inform regulatory measures.

Another aspect of EDC action is the ‘window of exposure,’ which refers to how the timing of EDC exposure during development may impact health outcomes. Traditionally, many of the risk-assessment studies of EDCs for female reproductive toxicity have been conducted in rodent models using a 28-day exposure model. However, in order to optimize toxicological testing methods, it is important to determine which developmental periods are most vulnerable to EDC exposure in order to design studies using animals at an age that optimizes sensitivity. In this Research Topic, Boberg et al. compared the effects of EDCs on female endocrine-dependent reproductive hallmarks in rodents during pre-pubertal exposure with those in adults. The authors hypothesized that early exposure may allow for a more sensitive assessment of reproductive toxicity due to the ability to include pubertal onset as an additional endpoint. In an analysis of two established EDCs, the estrogen diethylstilbestrol and the steroid synthesis inhibitor ketoconazole, reproductive-related outcomes such as estrous cyclicity, and ovarian follicular histology were found to be comparable regardless of life stage. The pre-pubertal cohort was sensitive to the acceleration of female pubertal reproductive hallmarks and the adult group displayed a more pronounced effect on non-reproductive organs such as the liver and adrenal glands. The authors suggested that 28-day *in vivo* toxicity studies with pre-pubertal animals are optimal for the detection of the reproductive system outcomes for agents with estrogenic activity, whereas non-reproductive organ function is best assessed in adult animals.

Non-alcoholic fatty liver disease (NAFLD) is the most widespread liver disease in humans and its prevalence is attributed in part to EDC exposure. In NAFLD, hepatocytes accumulate fat, with the presence of steatosis in >5% of hepatocytes being the criterion for diagnosis. Current toxicological assessment of steatosis via OECD protocols uses rodent models of EDC exposure with endpoints of *in vivo* liver histochemistry and liver function tests. In this Research Topic, Kubickova and Jacobs suggested that the development of alternative *in vitro* hepatocyte assays for chemical screening would be more time and cost-effective, and would provide mechanistic insights. Previous research observations allowed the authors to identify a list of suitable chemicals and controls for implementing and optimizing *in vitro* human steatosis *in vitro* testing methods in hepatocytes. A number of the selected chemicals are established EDCs, and several have known effects on PPARα and γ activities. The authors concluded that *in vitro* steatosis assays can be validated with these chemicals, and thus, endorse this convenient assay for screening of chemicals for human hepatotoxicity to inform regulatory measures going forward.

In conclusion, given the difficulty in assessing endpoints of exposure, it is beneficial to have new research on the molecular and structural effects of EDC exposures at various stages of life. The development of more sensitive EDC screening methods and toxicological assessments offers a valuable opportunity for prevention and/or intervention of the numerous global health problems related to EDC exposure, in particular obesity.

